# Thematic Reclassifications and Emerging Sciences

**DOI:** 10.1007/s10838-020-09526-2

**Published:** 2021-02-07

**Authors:** Raphaël Sandoz

**Affiliations:** Global Studies Institute, Unit of History and philosophy of science, University of Geneva, 30, Quai Ernest-Ansermet, 1205 Geneva, Switzerland

**Keywords:** Classification of the sciences, Emerging disciplines, History of disciplines, Thematic analysis

## Abstract

Over time, various thematic classifications have been put forward to organize science into a coherent system of specialized areas of research. From an analysis of the historical evolution of the criteria used to distinguish the sciences from one another, I propose in this paper a quadripartite typology for the different thematic classification systems propounded by scholars throughout the centuries. Basically, I argue that the criteria used to differentiate the sciences have been alternately drawn from their respective subject matters, kinds of knowledge, methods and aims. Then, I show that several reclassifications occurred in the thematic structure of science. Finally, I argue that such changes in the structure of learning displaced the modalities of contact between the objects, knowledge, methods and aims of the various branches of science, with the result of outlining reshaped intellectual territories conducive to the emergence of new areas of research.

## Introduction

In our world of information overload, organizing the thematic structure of science in a coherent way appears more important than ever. As a result, a marked interest in “knowledge organization” is evidenced in contemporary literature (Dahlberg 2006). The motivations behind such research are manifold. Faced with a proliferation of new themes and academic specialties, information scientists consider “thematic analysis” as an essential tool for data management and librarianship (Boyatzis 1998; Guest 2011). But beyond such practical goals, a reflection on the thematic structure of science is also highly relevant for philosophers, given the deep “philosophical underpinnings of knowledge organization” (Smiraglia 2014, 19). In this respect, the role of the thematic structure of science in scientific thought has been analyzed by several scholars (Holton 1975; Tondl 1998), while others have profitably inventoried the different “categories of scientific cultures” (Winther 2012) and the many “styles of scientific thinking” (Crombie 1994; Hacking 1994; Pickstone 2000). Additionally, the surge of interest in interdisciplinarity during the past decades has raised various philosophical issues regarding the impact of the disciplinary framework on the unity of science (Grantham 2004) and its influence on the dynamic of research (Stichweh 1990; Thorén and Persson 2013). Among the questions recently addressed in this context, some have wondered how changes in the thematic structure of science affect the emergence of new specialties (Wray 2005; Politi 2018). The Kuhnian concept of incommensurable “disciplinary matrices” (Kuhn 1969, postface) has played an important role in these discussions.

Obviously, knowing how the thematic structure of science has evolved over the centuries is an essential prerequisite for addressing such issues. However, while a scattered literature has examined separately the views of particular historical figures—such as Bacon or Comte—on the classification of the sciences, there are few comprehensive works aimed at analyzing the evolution of the thematic structure of science over an extensive period of time (apart from the rather descriptive studies of Flint 1904 and Kedrov 1965). As a result, changes in the classificatory criteria articulating the structure of science have never been systematically tracked. Such a purpose involves the adoption of a long time scale: “Periods illuminate each other […]. We need broad frames in which to think comparisons” (Pickstone 2000, 5). Although traditionally common, such an approach became out of fashion among historians since the second half of the twentieth century (Fisher 1990, 853). Even so, philosophers may have another stance on the past of science, as the research agenda of “historically informed philosophy of science” serves other purposes (Laudan 2016; Arabatzis 2017; Stadler 2017). In this respect, I argue that the debate on scientific change has much to gain from a reasoned historical overview of the evolution of the thematic structure of science.

With this in mind, I have compiled an online database, currently containing 150 classifications of the sciences put forward by a series of scholars from Antiquity to our times, systematically collected from historical sources.^1^ An analysis of this material reveals that, throughout the centuries, scholars have put forward various approaches to organize and classify scientific activities into a coherent thematic structure. Aristotle’s “Division of Theoretical Sciences” (treated in Cleary 1994), Lull’s “Arbor Scientiae” (1295), Reisch’s “Philosophiae partitio” (1503), Ramus’s “Tabula Artium” (1576), Bacon’s “Distribution of Human Knowledge” (1623), Bentham’s “Encyclopedic Table” (1816), Comte’s “Échelle encyclopédique” (1830), Ampère’s “Classification des connaissances humaines” (1834), Spencer’s “Classification of the Sciences” (1864) and Peirce’s “Detailed Classification of the Sciences” (1902) are only a few of the numerous attempts made over time to frame the various topics of research into a *system* (Atkins 2014). A review of the evolution of such thematic classifications over an extensive historical period reveals considerable variations in the approaches to the organization of research. It is not merely that, as one might easily expect, the list of disciplines mentioned in historical sources is ever changing. A transformation also occurred at a deeper level: the *types of criteria*^2^ used to differentiate the sciences from one another have substantially changed over time. Operating as a logical framework, “each classification calls for the application of a system of classification criteria” (Tondl 1998, 245). Any change in the principles underlying such classificatory schemes has far-reaching implications, that extend beyond simply redrawing the disciplinary borders. The main purpose of my paper is to examine the influence of such thematic reclassifications on the advent of emerging fields of research.

In the next section, I introduce a quadripartite typology—intended to be operative in history—of four classificatory paradigms used by scholars to organize the various areas of research into a coherent system. I will show that the sciences have been successively distinguished from one another according to their subject matter, the kind of knowledge they involve, their methods, and their respective purposes. In Sect. 3, I discuss whether and how these four types of classification can be combined with one another. This will lead me to highlight their orthogonality. In Sect. 4, I analyze how the classificatory criteria underlying the thematic structure of science evolved over the centuries. In Sect. 5, I show that several thematic reclassifications (i.e., disciplinary paradigm shifts) occurred over time. Finally, I argue that such global changes in the organization of science involve logical constraints, entailing recombinations of the disciplinary clusters, which foster the emergence of new scientific fields.

## Classifications of the Sciences: A Typology

Since ancient times, scholars have always been interested in the logical principles underlying the organization of science (Blair 2007), so much so that philosophy has sometimes been assigned the function of “a science of the sciences which determines the principles and conditions, the limits and relations, of the sciences” (Flint 1904, 3). The importance of this “architectonic function” (Atkins 2014) of philosophy has varied in intensity over time. Echoing our contemporary motivations in this respect, it was often when faced with information overload that scholars felt the need to rethink the thematic structure of science. Following the rise of printing in Modern Europe, an unprecedented proliferation of books raised “challenges of managing overabundant information” (Blair 2010). In order to cope with this massive influx of new publications, libraries had to be reorganized in a way that their readers could find the relevant information they were looking for, resulting in an increased interest in the classification of the sciences in the early modern period (Freedman 1994). During the nineteenth century, the growth of specialization involved a reorganization of scientific practices, whence an exceptional interest in thematic classifications. In addition to such purposes of managing libraries and research activities, the systematic mapping of the fields of learning was often aimed at the identification of uncharted scholarly territories still to be explored. Various approaches to the classification of the sciences have been put forward over time, which I will now examine.

### Ontological Classifications of the Sciences

The most direct and traditional way to compartmentalize science into distinct areas of research is to distinguish them from one another according to the *objects* they study. Such classifications of the sciences by *subject matter*—which I call *ontological*—have been put forward by numerous scholars since ancient times, such as Aristotle, Boethius, Cassiodorus, Avicenna, Kilwardby, amongst many others. The tremendous influence of Aristotle makes him the undisputed historical leader of this mode of classification. His disciplinary structure, based on categories organized in a system of *genera*, *species* and *differentia*, is archetypal of a classification of the sciences by subject matter: “every science has its own subject-genus which is assumed to exist and is defined at the beginning of an inquiry, which then demonstrates that certain per se attributes belong necessarily to that subject” (Cleary 1994, 34). Divisions between the Aristotelian fields are drawn from the very nature of the objects under study in each area of learning. A first classificatory criterion opposes *natural* to *artificial* entities, underlying a crisp distinction between the study of nature (theoretical philosophy) and technological arts handling mere artifacts (τεχνητόν). Theoretical philosophy is further divided into “three branches of study, one of things which are incapable of motion, the second of things in motion, but indestructible, the third of destructible things” (*Phys*. 198a30–31). Those fields are, respectively: i. metaphysics, dealing with “substantial universals” (Perin 2007), ii. mathematics, which involves “intelligible matter” (Gaukroger 1980), and iii. physics, concerned with “corruptible matter”. In addition, the realm of physics (limited to sub-lunar terrestrial matter) is ontologically distinguished from the sphere of study of astronomy, dealing with the supra-lunar world.

Aristotle’s ontological divisions, diagrammatically portrayed as a dichotomic tree in Porphyry’s *Isagoge*—a text meticulously translated into Latin by Boethius in the early sixth century—exerted a considerable and lasting influence on the medieval world. For instance, the “Divisio scientiae” put forward by Robert Kilwardby in his *De ortu scientiarum* (1279) takes up the Aristotelian system of classification by subject matter (Sharp 1934, 6; Alessio 2001, 108–109; Maierù 2012, 359): “science must be divided according to the division of things about which it is” (Kilwardby 1279, III.5). Each field has its own specific subject matter: arithmetic is about numbers (Kilwardby 1279, XIX.135), geometry deals with proportions in figures (Kilwardby 1279, XI.61), astronomy with heavenly bodies (Kilwardby 1279, XII.68), and so on. Similar disciplinary schemes appear in the *Didascalicon* (1130) of Hugh of Saint-Victor, and in Arnoul of Provence’s “Divisio scientiarum” (1250). In fact, most medieval classifications of the sciences were based on the recurring adage: “tot scibilia quot scientiae”, which means: “as many sciences as there are objects of science” (Bermon 2018, 302).

### Epistemological Classifications of the Sciences

Another and more subtle way to classify the sciences—which I call *epistemological*—is based on the very *nature of the knowledge* involved in each field. Such an approach has been propounded by many scholars of the sixteenth and seventeenth centuries: in *De disciplinis* (1531), Juan Luis Vives devised a classification of the sciences based on the “divisions of different kinds of knowledge”. A similar partitioning scheme appears in Juan Huarte’s *Examen de ingenios para las Sciencias* (1575), taken up by Pierre Charron in the first book of *De la Sagesse* (1601), and by Antonio Zara in his encyclopedia *Anatomia ingeniorum et scientiarum* (1615). But it is especially Francis Bacon who crystallized the principles of such an epistemological approach in his original “tree of knowledge” departing explicitly from the Aristotelian tradition. Rather than categorizing the sciences by subject areas, Bacon organized the different fields of learning according to a “taxonomical structure of knowledge […] derived from an epistemological viewpoint” (Klein 2004, 70). More specifically, the classification advocated in *The Proficience and Advancement of Learning* (1605) demarcates the various territories of learning from one another according to the kind of knowledge each of them involves (Kusukawa 1996, 48). An epistemological classificatory structure further developed in his *Descriptio Globi Intellectualis*: “I adopt that division of human learning which corresponds to the three faculties of the understanding. History is referred to the Memory; poesy to the Imagination; philosophy to the Reason” (Bacon 1612, I). Such a cognitive categorization introduces an important distinction between the philosophical sciences (dealing with knowledge acquired by the intellectual faculties of Man), and the historical sciences (collecting empirical facts perceived by senses). The word “history” being potentially misleading for a contemporary reader, its meaning requires further clarification: while “civil history” provided knowledge related to the (present and past) deeds and works of men, a significant part of history dealt with the study of nature, under the label of “natural history” (Manzo 2012, 34). This later branch of learning aimed at collecting, through empirical observations, knowledge about natural phenomenon regarding Earth, sea, meteors, elements, living species, and so on. That is to say, the various sciences of nature were distributed between two main epistemological sub-divisions: “natural philosophy”, relying on rational demonstrations to establish the general laws governing the world, and “natural history”, aimed at collecting sensory knowledge about particular facts trough an empirical approach.

The Baconian disciplinary system exerted a strong influence on the early modern world. In this respect, “the seriousness with which the early Fellows of the Royal Society attempted to implement Bacon’s program for the reform of knowledge in their collective activity” (Lynch 2005, 173) has been convincingly established. Purver insists on the foundational role of Bacon’s classification in the process of replacing the antiquated Aristotelian disciplinary system: “Society’s central purpose—putting into action the vision of Francis Bacon—was to cut away the whole existing system of natural sciences, and deliberately to begin the process of creating an organized body of knowledge” (Purver 1967, 235). Moreover, the Baconian “epistemological strategy” (Darnton 1984) offered the Encyclopedists an articulated structure implemented in Alsted’s *Encyclopaedia* (1630), Chambers’ Cyclopedia (1728), and most notably in the *Prospectus de l’encyclopédie* (1750), where Diderot used straightforwardly the Baconian map of knowledge (Vert 1994, 357).

Although less remarked than the Baconian “general distribution of human knowledge”, the classification of the sciences put forward by Hobbes in the *Leviathan* offers another archetypal example of an epistemological disciplinary system. Like Bacon, he distinguishes the branches of learning according to the nature of the knowledge they involve: “There are of knowledge two kinds, whereof one is knowledge of fact; the other, knowledge of the consequence of one affirmation to another. The former is nothing else but sense and memory, […] and this is the knowledge required in a witness. The latter is called science, […] and this is the knowledge required in a philosopher; that is to say, of him that pretends to reasoning.” (Hobbes 1651, IX, 51). The knowledge of facts (indiscriminately present or past) is served by senses and memory, and belongs to history. On the other hand, knowledge produced by reason is the special realm of philosophy (Hobbes 1651, IX, 51). Its various subfields are further divided according to a very coherent epistemological scheme, actually more self-consistent than the baconian system (Flint 1904, 118).

### Methodological Classification of the Sciences

A third approach to the thematic organization of science is to differentiate the various areas of learning according to the *methods* specific to each field. This classificatory paradigm—which I call *methodological*—has been sustained by several scholars over time, such as Condorcet, Destutt-Tracy, Candolle, Coleridge and many others. Rejecting the Baconian division of science according to the faculties of the mind, Condorcet points out that “there is no science in which understanding, imagination and memory are not involved together”.^3^ Advocating “a methodological classification of the sciences” (Crépel and Rieucau 2005, 269), he prefers to “classify the sciences according to the methods they use.”^4^ A list of the different methods underlying each science is given in the *Tableau général*, further developed in a draft manuscript of his *Tableau historique* (1793c) entitled “Sur les méthodes techniques”. Condorcet’s list of methods includes *analysis*; *observation*; *measurement*; *experimentation*; *deduction*; *hypothetical reasoning*; *analogical reasoning*; *detection of anomalies*; *combinations* and *the use of probabilities*. These ten methods were at the base of a combinatorial system of divisions, which can be seen as an anticipation of Dewey’s decimal classification (Baker 1962, 100). According to such a system, two distinct sciences differing in their respective methods can share the same subject matter. For instance, a chemist and a mineralogist can study the same rock, but using different methods.^5^

This type of classification paradigm became very common during the first half of the nineteenth century. Coleridge’s *Preliminary Treatise on Method* (1818)—serving as an introduction to the *Encyclopaedia Metropolitana*—offers another typical example of a methodological system, entitled “Methodical compendium of Human Knowledge” (Coleridge 1818, 2). In order to capture the relevant connections between things, each science has to rely on a specific method: “As soon as the mind becomes accustomed to contemplate, not *things* only, but likewise *relations* of things, there is immediate need of some path or way of transit from one to the other of the things related […] in short, there must be Method.” (Coleridge 1818, 2–3). Deductive sciences, such as mechanics or mathematics, establish laws of necessity between causes and effects. An operation directed by some specific “processes of reasoning” (Coleridge 1818, 35). Relations between the qualities of things are investigated by “observation, aided by experiment” (Coleridge 1818, 11). This is the method of empirical sciences: “Medicine, Chemistry, and Physiology, are examples of a Method founded on this second sort of relation” (Coleridge 1818, 3). The method proper to Fine arts is another one: “Between these two, lies the Method of the Fine Arts, a Method in which certain great truths, composing what are usually called the *laws of taste*, necessarily predominate” (Coleridge 1818, 3). In every science, a method implements “some rule, some mode of union, more or less strictly necessary.” (Coleridge 1818, 4).

### Teleological Classification of the Sciences

A fourth possible approach to the thematic structure of science is to differentiate its various fields of research according to their specific *purposes*. Over time, such a classificatory paradigm—which I call *teleological*—has been repeatedly propounded by numerous scholars. Divisions implemented in Jeremy Bentham’s “Encyclopedical tree” categorize the sciences according to their “end-in-view” (Bentham 1816, table V). For instance, the criterion used to distinguish geography from geognosy is the following: “By *Geography*, the Earth is considered with a view to one *set of purposes*; by *Geognosy*, with a view to *another* set of purposes” (Bentham 1816, 40). Dependencies between the respective aims of the different sciences are further hierarchized through a succession of dichotomic bifurcations opposing pairs of disciplines on the basis of their purposes. That way, relying on the objectives to be attained in each particular science (Machlup 1982, 62), Bentham’s disciplinary scheme is explicitly based on teleological classificatory criteria.

Amongst other such teleological classifications of the sciences, it is worth mentioning the *Geno-grafia dello Scibile, considerato nella sua unità di utile e di fine* (1829) of the Neapolitan professor Giacinto de Pamphilis, who also attempted to assign a specific “function” to each discipline. In a similar way, the *Classification des connaissances humaines* of the Belgium naturalist Jean-Baptiste d’Omalius d’Halloy is explicitly based on teleological criteria: “It seems rational to divide science according to the goals pursued in each particular field of research.[…] Groups obtained by means of that criterium are much more natural than those formed on the basis of the faculties of the mind or on the nature of knowledge.” (d’Omalius d’Halloy 1834, 2).^6^ Adrien Naville, “Professor of logic, methodology and classification of the sciences” at the University of Geneva at the turn of the twentieth century, offered another archetypal example of a teleological classificatory paradigm: “Sciences provide sets of answers to questions asked by the human mind. As such, the deepest distinctions between the sciences lie in the differences in the questions being asked” (Naville 1901, 11).^7^ Each particular science aims at answering specific questions; as such each field of research “reflects the choice of a specific purpose” (Naville 1901, 150). Naville divides scientific fields into three main groups, pertaining respectively to the three following questions: theorematic sciences aim at answering the question “what is possible?”, historical sciences are concerned with the question “what is real?”, and normative sciences with the question “what is good?”. Particular fields are then further subdivided according to their various purposes.

Last but not least, one of the most prototypic *teleological* classification of the sciences has been put forward by Charles Sanders Peirce, whose disciplinary system “delimits the aim of each science and states its authority over or subjection to the other sciences” (Atkins 2006, 497). According to his views, each scientific field is centered on a specific research question: “a science is defined by its problem” (Peirce 1902, 1.127). As such, each field is distinguished from the other ones by its specific *purpose*: “division of science will be according to its fundamental purpose, making what I shall term branches of science. A modification of a general purpose may constitute a subbranch” (Peirce 1902, 1.238). A sophisticated logical apparatus of *branches*, *classes*, *orders* and *families* is used to further organize the various fields of research according to their aims.

To sum up, the historical examples discussed in this section exemplify four contrasting kinds of criteria, alternately used over time to underlie the thematic structure of science. The various areas of research can be compared against each other on four significantly different classificatory paradigms, handily characterized in the following typology:

- 1. *Ontological classifications*: scientific fields are distinguished from one another according to the kind of *objects* they study (classification by subject matter);
- 2. *Epistemological classifications*: scientific fields are distinguished from one another according to the kind of *knowledge* they involve;
- 3. *Methodological classifications*: scientific fields are distinguished from one another according to the kind of *methods* they use;
- 4. *Teleological* (or *goal-related*) *classifications*: scientific fields are distinguished from one another according to the kind of *purposes* they pursue.

While extracted from historical material, my typology appears to be in line with the thematic analysis put forward by Tondl (1998). Indeed, he explicitly acknowledged all four categories listed above: (1). “each discipline has its own subject of study […], i.e. a set of entities, objects, phenomena or events which fall into the competence of the given discipline.” (Tondl 1998, 260); (2). “a scientific discipline presupposes the existence of […] a body of knowledge” (Tondl 1998, 262); (3). “each scientific discipline is connected with a certain *complex of methods”* (Tondl 1998, 261) and (4). “As far as each discipline is concerned, there is a characteristic *complex of goals*” (Tondl 1998, 261). Such a typology refers to four types of constituents necessarily involved in any discipline. Indeed, each science has a *purpose*, uses some *methods*, in order to acquire *knowledge* about some *subject matter*.

## Orthogonal Classificatory Types

An important question arises regarding the compatibility of the classificatory types listed in the above typology: are they mutually exclusive or not? To put it another way, is it possible to establish a thematic classification combining several criteria drawn from more than one of the four categories listed above? Answering this question requires determining whether a one-to-one correspondence exists between the *objects*, *knowledge*, *methods* and *purposes* of the different sciences. Obviously, a classification by subject matter for example, can hardly be expected to coincide with a classification by methods, as the methodological categories would most likely overlap with the ontological ones. To use a geographical metaphor, linguistic boundaries rarely coincide with the political ones, and a similar phenomenon is expected with the disciplinary borders established according to different classificatory types. Thus, it may seem impossible to combine several types of criteria within a single thematic classification. And yet, historical sources reveal that some scholars have used to that end more than one type of criteria. In this respect, two cases must be distinguished:

- *Incoherence*: a scholar puts forward (typically, in separate publications) several *unrelated* classifications of the sciences, each of them being based on a different type of criteria. For instance, an early manuscript presents an ontological system, while a treatise written at the age of maturity propounds an epistemological classification. In such a case, this may simply reflect an evolution in the author’s thinking, without any logical commitment.
- *Multi-dimensional* system: a scholar attempts to combine together criteria of different types in a single classification. Depending on the case, the outcome of such attempts can be more or less convincing from a logical point of view.

While a mere coexistence of separate instances of different classificatory types is not very telling about their mutual compatibility, attempts to build *multi-criteria* classification systems is way more interesting in this respect. I will focus now on two such cases of classifications combining criteria drawn from different types. Contrariwise to the examples mentioned in the last chapter, propounded by influential figures, the two classifications of the sciences I will discuss here were developed by relatively unknown scholars: Louis Bourdeau’s *Plan de science intégrale* (1882), and George Albert Cogswell’s *Classification of the sciences* (1899). Although they were not powerful enough to exert a significant influence on the evolution of the disciplinary landscape, their classification systems are worth being studied as interesting “extreme cases”. Both of them attempted to combine ontological and methodological criteria to classify the sciences, but while the former *parallelized* objects and methods, they were considered as sharply *orthogonal* by the latter.

**Fig. 1:**
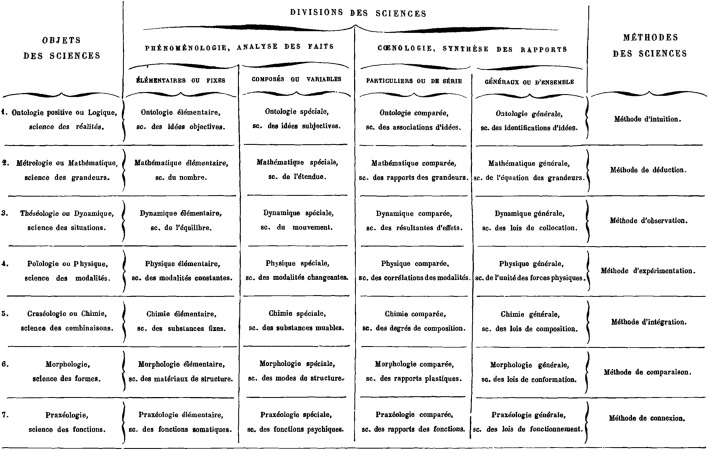
Louis Bourdeau’s “classification des sciences” (1882)

The “division des sciences” (Fig. 1) put forward by the French historian and philosopher Louis Bourdeau in his *Plan de science intégrale* (1882) classifies scientific fields in a table based on both *ontological* and *methodological* criteria: “Sciences differ by their objects and methods” (Bourdeau 1882, iii). As per Fig. 1 below, it appears that the first column of his table, entitled “objects of the sciences”, is parallel to the last one, entitled “methods of the sciences”. That is to say, Bourdeau postulates the existence of a *one-to-one correspondence* between objects and methods, as explicitly stated in the following passage:

Each science is constrained, by the very specialty of its subject matter, to explore it by means of an appropriate research mode. Such an investigation procedure is called its *method*.[…] Considerations leading to the division of natural facts in categories also lead to such a categorization in the manners to studying them. As a result, it is necessary to institute as many methods as there are sciences. (Bourdeau 1882, I, 36–37).^8^

One method per subject matter, and one subject matter per method: such is the main principle underlying Bourdeau’s classification. As a result, no science can use more than one unique method: “A change of method can never occur, as no science has several methods at its disposal” (Bourdeau 1882, I, 45). Moreover, the method of a particular science can never be used by another science: “this is not a question of degrees, but of *exclusive* use” (Bourdeau 1882, I, 344). That is to say, classifications by objects and by methods are explicitly parallelized: “we have shown the parallelism of such divisions of the sciences” (Bourdeau 1882, I, 50).

According to such considerations, Bourdeau claims that each science investigates its own subject matter using an exclusive method. *Deduction* is the strict prerogative of mathematics, and cannot be used in any other science. This prevents deduction from playing the slightest role in logic, for instance, which must be based on a different method: intuition. *Experimentation* is for the exclusive use of the physicist in the exploration of material phenomena. Therefore, being specific to physics, this method is irrelevant in chemistry, which relies on another method: integration. Does this mean that chemistry is not an experimental science? As unbelievable as it sounds, this is what Bourdeau explicitly asserts: “Experimentation, so efficient for the investigation of physical modalities, is useless to the understanding of chemical changes” (Bourdeau 1882, II, 289). Actually, chemists are not even supposed to observe anything, as the method of *observation* is the strict prerogative of the science of dynamic: “Observation appears no more relevant to study chemical phenomena, as atoms are even less visible than are molecules” (Bourdeau 1882, II, 289). As for the method of *comparison*, morphology alone is allowed to use it: “Only morphology may apply the method of comparison” (Bourdeau 1882, II, 446).

No need to go any further in the presentation of Bourdeau's system to realize how unsatisfactory it is. The surprising views to which his classificatory principles have led him (such as regarding chemistry as a non-experimental science) appear of course incredibly unsound. However, despite its ineptitude, this failed attempt at combining ontological and methodological classificatory criteria teaches us something important, which is not immediately obvious: it highlights straightforwardly the lack of a one-to-one correspondence between the objects and methods of science. Inadvertently, Bourdeau provided us with a convincing *reductio ad absurdum* in this respect.

The main problem with Bourdeau’s disciplinary scheme lies in his misguided attempt to combine *orthogonal* criteria. Classifications of books in libraries offer a good metaphor to understand the issue. A librarian can choose to index a collection of books *either* alphabetically by author names, *or* chronologically by year of publication. But it is totally impossible to have both: ordering books chronologically will break the alphabetical order, and vice versa. Of course, the librarian can draw up two *distinct* catalogues, each of them based on a single criterion, but it is not possible to make a linear index respecting both the alphabetical and the chronological order, because no one-to-one correspondence can be established between author names and publication dates. The same applies to the classifications of the sciences: as there is no one-to-one correspondence between the objects, knowledge, methods, and aims of the various fields of research, grouping the sciences by subject matter will break methodological categories, and conversely. Therefore, it seems difficult to build a sound multi-criteria classification along the lines of Bourdeau’s principles.

**Fig. 2:**
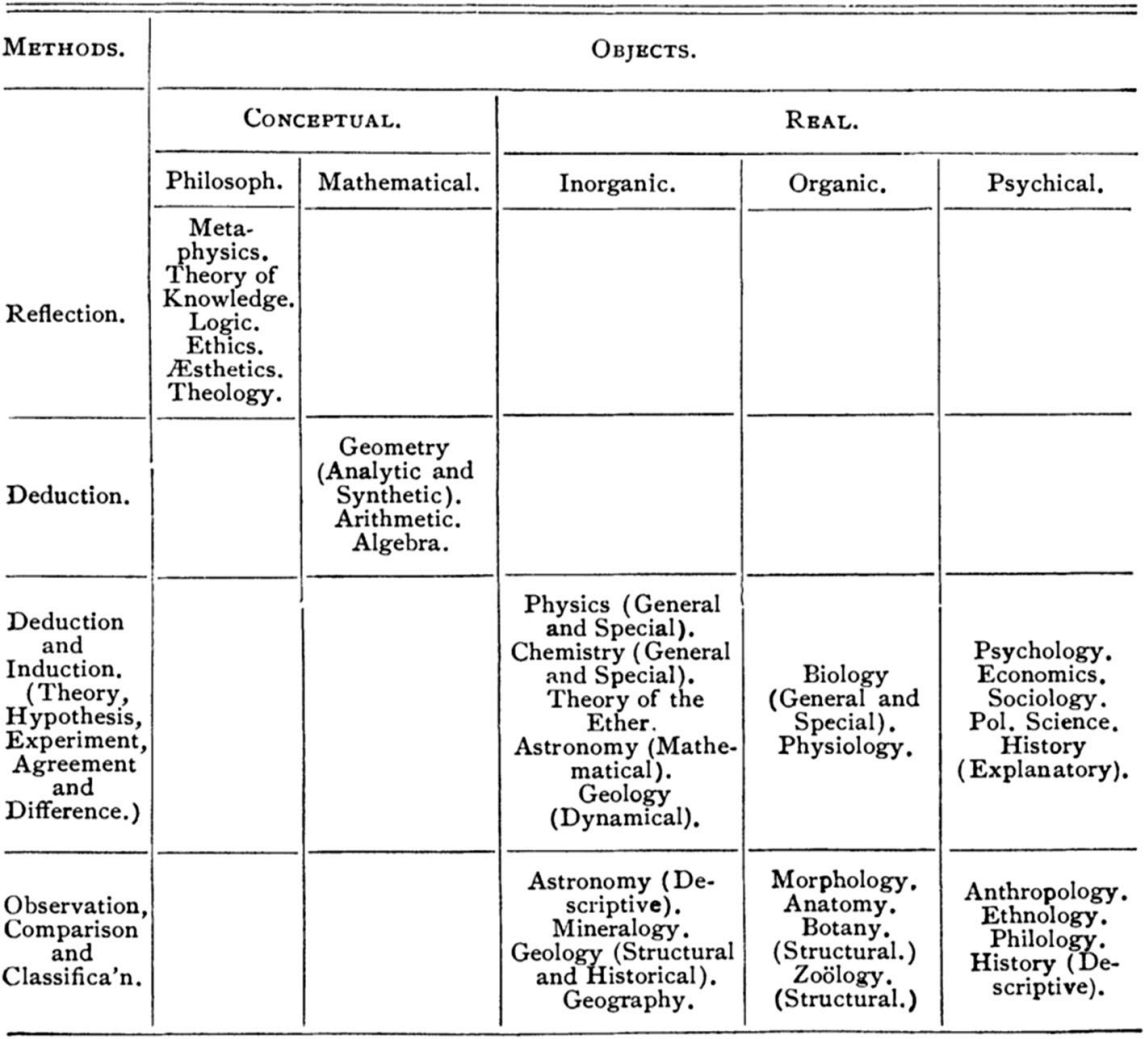
Cogswell’s classification of the sciences (1889)

However, other approaches are possible. The perfect antithesis of Bourdeau’s system has been sustained by the Canadian scholar George Albert Cogswell.^9^ In his classification, the objects and methods of the sciences are not parallel, but *orthogonal* to one another, as appears explicitly on Fig. 2 below. That way, Cogswell acknowledges, in the logical form of his classificatory system, that there is no one-to-one correspondence between the objects and methods of the sciences. In this respect, he points out that it is common for a method to be relevant for the study of various subject matters, and as such to be shared amongst several disciplines: “the same method may be applicable to widely different spheres of the real world” (Cogswell 1899, 504). Conversely, the analysis of a given object often requires multiple methods. In order to account for these overlaps, both objects and methods must be factored in when establishing a classification of the sciences:

In constructing a plan of classification upon ultimate principles, it would seem that two distinct factors must be given equal consideration.[…] A division of the sciences based upon method needs to be supplemented by a division based upon the nature of the subject-matter upon which it is employed. (Cogswell 1899, 504–505).

But if no one-to-one correspondence exists between the respective subject matters and methods of the sciences, how is it possible to combine these criteria in a relevant way? Cogswell’s answer is to reject linear order in favor of a *two-dimensional* system: “a true scheme of classification should be explicitly two-dimensional, having reference both to […] subject matter and method” (Cogswell 1899, 506). That way, *intersections* between objects and methods are acknowledged, increasing the flexibility of the classification. In such a scheme, any method can potentially (but not necessarily) be used for the investigation of any subject matter. As a result, most limitations of Bourdeau’s system disappear: for instance, the method of comparison (last line of Fig. 2) is no longer limited to morphology alone, but can also be used in other sciences of the inorganic world (such as astronomy, mineralogy and geology), as well as in various human sciences (anthropology, ethnology, philology and history). What is more, several cells in Cogswell’s table are left empty. That is to say, there is room for additional sciences not yet established, obtained by combining together different methods and subject matters. As a result, Cogswell’s table possesses a heuristic character, potentially useful to discover new disciplines. I will elaborate on that mechanism in Sect. 4.

Even if Cogswell’s table is not flawless, its underlying principles are undoubtedly much more sound than the approach propounded by Bourdeau. Several scholars have put forward similar classificatory systems, sometimes involving the other types of criteria listed in my typology. For instance, Isidore Geoffroy Saint-Hilaire has developed a two-dimensional classification aimed at combining ontological and teleological criteria: “the classification of the sciences can be reduced to a *double-entry table*. Sciences analogous by their objects are to be placed on a same *horizontal* line, while sciences that are similar by their aims will be on a same *vertical* column.”^10^ (Saint-Hilaire 1854, I, 261). Furthermore, nothing prevents these tabular devices from using more than two axes: a classification including all four types of criteria would be tabulated in a four-dimensional system of orthogonal axes. Although hard to be drawn graphically, this is logically unproblematic. Among the scholars who have propounded a multi-dimensional classification based on more than two types of criteria, it is worth mentioning Cournot (1851), Dove (1851), Wilson (1856) and Bain (1870).

The most important result reached here is that the criteria underlying the four classificatory types enumerated in the typology developed in Sect. 2 are largely *independent* of each other—or even *orthogonal*, as displayed on Cogswell diagram (Fig. 2). In Kuhnian words, we might consider these four classificatory paradigms to be “incommensurable” with each other. This may appear counter-intuitive, as one could wrongly expect, like Bourdeau, the purpose of each science to determine univocally a specific method, expressly required to acquire peculiar knowledge pertaining to its exclusive subject matter. Admittedly, a particular method can prove more efficient than another one to address the questions and problems of a specific discipline. Some tools may appear, at first sight, peculiar to a specific science, and as such irrelevant to all others. A microscope, for instance, first seems to be useful exclusively for the observation of small objects, and thus completely useless for an astronomer studying huge stars or planets. And yet, Pluto was discovered with a microscope, used to magnify photographs of the sky (Tombaugh and Moore 1980, 11). Such crossovers between the objects, methods and aims of the different sciences are very frequent. Therefore, it is not tenable to assume a one-to-one correspondence between the elements of the four classificatory types listed above, as made clear by Bourdeau’s unintended *reductio ad absurdum* in this respect. However, the recourse to intersectional logical devices, such as Cogswell double-entry table, offers a path to the establishment of multi-dimensional classificatory systems. But it also evidences a tension between incompatible—or *orthogonal*—types of criteria, resulting in logical constraints, which I will discuss later in Sect. 5.

## Thematic Reclassifications

Now that I have analyzed the different types of criteria used in history to distinguish the sciences from one another, as well as the modalities of their combination, the next question I will address concerns their evolution over time. The idea that the disciplinary structure of science undergoes, at times, significant overhauls has been considered by several scholars. Kuhn is known to have put forward, in the postscript of the *Structure*, the concept of “disciplinary matrix” as a substratum for paradigm shifts: “All or most of the objects of group commitment that my original text makes paradigms, parts of paradigms, or paradigmatic are constituents of the disciplinary matrix” (Kuhn 1969, 182). Admittedly, his approach was centered on single disciplines, understood as the primary framework for paradigm shifts, while mine is based on the whole disciplinary structure. Clearly, the criteria used to distinguish the sciences from one another in a thematic classification differ from the components of a disciplinary matrix, so that I cannot follow the Kuhnian line in a strict way. Even so, his concept of “paradigm shifts” can be profitably extended to approach significant overhauls in the criteria used to classify the sciences over time. Once replaced with the more accurate wording of “thematic reclassification”, this notion proves particularly useful to capture structural modifications in the disciplinary landscape. With this in mind, I will now examine if changes occurred over time in the types of criteria used to classify the sciences.

In order to have a quantitative idea of the distribution of the classificatory types over time, I have performed a statistical treatment of the data contained in my above-mentioned *Interactive historical atlas of the disciplines*. Clearly, it would be unfeasible to present here an in-depth account of each of the 150 thematic classifications currently contained in my database. But anyway, developing a broad and unfocused narrative of the twenty-five centuries of history it covers would be of little use for my purpose. Rather, it is the ability of studying accurately the evolution of narrowly defined parameters that my research requires. To this end, I have implemented several analysis tools in my database, which I will use in support of my argument, knowing that the raw historical material on which my computations are based is available online to my readers.^11^ In particular, I relied on the “advanced filters” feature to calculate the proportion of disciplinary maps of each type (ontological, epistemological, methodological and teleological) for a succession of time intervals. This approach should accurately reflect the evolution of the classificatory types over the centuries.

**Fig. 3:**
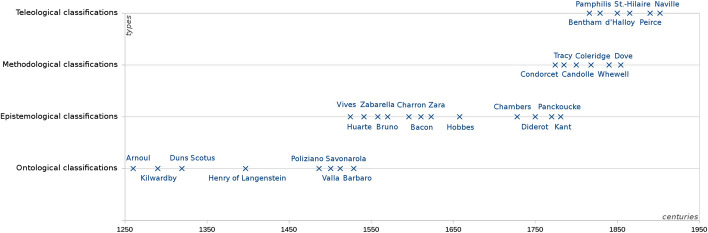
Chronological distribution of the classificatory types

A systematic review of the abundant historical material contained in my database places Vives among the first scholars to have explicitly adopted an epistemological classification of the sciences, in 1531. The first methodological disciplinary system to appear, in 1793, seems to be the one of Condorcet, which I have presented above. And the first to be based on teleological criteria is the one of Bentham, in 1816. Limiting my analysis to the classifications contained in my database that are underpinned by clearly defined criteria, I have calculated that 87% of those published between 1531 and 1793 involved epistemological criteria, while none of them used methodological ones. Between 1793 and 1816, no less than 80% of the classifications involved methodological criteria, while none of them had recourse to teleological ones. After Bentham, in contrast, 53% of them were based on teleological criteria. To put it another way, while traditionally ontological, a significant proportion of the classifications of the sciences shifted to epistemological criteria around the mid-sixteenth century, this type becoming prevalent until the end of the eighteenth century, when methodological criteria started to be systematically used. Since the first part of the nineteenth century, the adoption of teleological classificatory criteria increased substantially. Regardless of the number of criteria involved in the classifying process, if we examine when each of the four types have *emerged* in the classifications over the centuries, it is markedly apparent that they were introduced successively in the following order: ontological → epistemological → methodological → teleological. Such a trend is evidenced in the Fig. 3 below, charting the distribution of all the single-type classifications I have identified in my database for the period 1250–1950:

Clearly, whenever a new classificatory type emerged, the older thematic structures were unlikely to disappear overnight, resulting in a coexistence of different systems at certain epochs. On Fig. 3, an overlap between ontological and epistemological classifications is evidenced in the first half of the sixteenth century, while during the nineteenth century, methodological and teleological systems coexisted for a couple decades. Therefore, with the passage of time, a increasing number of scholars became tempted to combine together several classificatory types, as attempted by Bourdeau and Cogswell for instance. According to my database, only 11% of the thematic classifications published before 1600 included more than one type of criteria, compared to 25% between 1600 and 1800, and no less than 59% since 1800. However, due to their *orthogonality* (discussed in the last section), these four types of criteria cannot be combined straightforwardly. As a result, it became increasingly necessary to use combinatorial devices, such as Cogswell’s *double-entry array*. A look at the iconographic database of the *Interactive historical atlas of the disciplines* reveals that the use of such tables for classifying the sciences significantly increased during the nineteenth century.

To take up my comparison with a library, it is frequent to find books with several call numbers, each of them added to the former ones during thematic reclassifications. Recent books are indexed according to current classificatory principles, but the librarian will probably decide not to erase the old call numbers in order not to break potential references to the previous system. Most certainly, that will come with the need to establish a conversion *table* to cross-index the successive versions of call numbers. I argue that a similar process occurred every time the thematic structure of science was substantially reorganized: new classification systems were added to, rather than substituted for, the former ones. However, as discussed above, no one-to-one correspondence can be established between their respective elements. As a result, they cannot be combined straightforwardly, but only through combinatorial schemes such as *multiple-entry tables*. In this respect, as explained in the last section, “empty cells” were often identified when such arrays were built, as was the case in Cogswell’s chart (Fig. 2): his crossing of a list of subject matter with a—literally orthogonal—list of methods revealed several unexplored combinations. Of course, it is compelling for scholars to explore (implicitly or explicitly) such untapped intellectual territories. Especially as new disciplines are likely to emerge in such cross-reclassifications spaces.

## Recombinations and Emerging Sciences

While one might expect that the advent of new sciences will foster thematic reclassifications, the reverse process is harder to comprehend. Nevertheless, I argue that the emergence of a new area of research may be necessarily induced by a thematic reclassification, mainly because of the *logical constraints* involved in such a process. Admittedly, it would be dubious to claim that any scholar had enough power to dictate authoritatively the creation of a new discipline through a prescriptive reclassification of the sciences. Although several famous figures, such as Aristotle or Bacon, have been very influent in this respect, most plans of individual scholars to restructure the organization of research did not succeed. Ampère, for instance, failed to introduce in the disciplinary landscape the new fields (such as “arithmology” or “megethology”) planned in his classification. And yet, several thematic reclassifications have indeed occurred over time, even if rarely motivated by the wishes of individual scholars alone. For instance, in the climate of increased specialization of the nineteenth century, it proved appropriate to reorganize the division of work according to the various *skills* mastered by researchers. To this end, the adoption of a methodological classificatory system came about quite naturally, with in its wake the introduction of new disciplinary categories, such as the opposition between “theoretical” and “experimental” science, or the distinction between “pure” and “applied” research (Roll-Hansen 2017). That is to say, a change of classificatory paradigm is rarely planned by individuals, but results from a wider sociological context. However, every classification implements a constraining logical structure. As a result, any thematic reclassification also involves *logical constraints*, especially *combinatorial* ones.

**Fig. 4:**
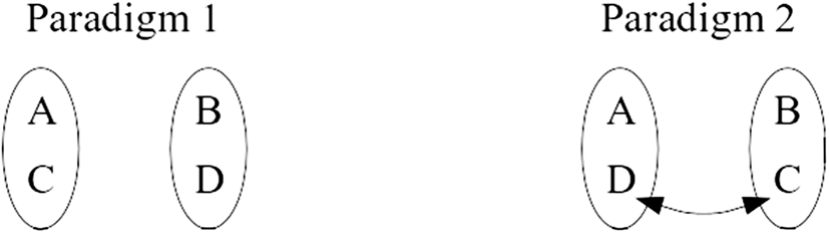
Permutations after a change of classificatory paradigm

These combinatorial constraints will best appear in the following example. Let us consider four musicians: a German pianist (A), an English violinist (B), an English pianist (C) and a German violinist (D). According to a “musical classificatory paradigm”, they can be divided into two groups defined by their instruments: the first one bringing together the pianists A and C, and the second one including the violinists B and D. Quite the opposite, the groups formed according to a “linguistic classificatory paradigm” will include: i) the German-speakers A and D, and ii) the English-speakers B and C. Consequently, changing the classificatory paradigm involves the permutation of the musicians C and D between the groups (Fig. 4). As a result, the *modalities of contact* between A, B, C and D are transformed. This will introduce new interactions: adopting a musical paradigm will bring together people speaking different languages, so that they will have to acquire linguistic skills in order to understand each other. By contrast, according to a linguistic paradigm, they will rather need to learn how to play piano and violin together. Such a combinatorics is subtended by the lack of a one-to-one correspondence between languages and musical instruments.

I argue that similar recombinations inevitably occur when the objects, knowledge, methods and aims of the sciences are redistributed into new disciplinary categories in the aftermath of a thematic reclassification. Depending on the classificatory paradigm adopted, the differentiation of disciplines involves forming sets of objects, knowledge, methods, or aims. But as repeatedly mentioned, there is no one-to-one correspondence between the elements of such sets. As a result, during a thematic reclassification, at least some of the elements of these four categories will be *displaced*, as was the case for our musicians C and D above. It means that a change of classificatory paradigm *generates new interactions* between the objects, knowledge, methods and aims of science. Clearly, this can reorient research in innovative directions. Such a mechanism will become clearer on a concrete example: the emergence of *mixed mathematics* at the turn of the seventeenth century.

By the end of the Renaissance, a variegated group of scholars launched an innovative area of research referred to as *mixed mathematics*, based on an interplay between mathematical reasonings and empirical practices emanating from various “mechanical arts”.^12^ Actually, this appellation hides one of the most direct ancestors of the mathematized physics we are accustomed to: “one might conveniently see *mixed mathematics* as modern physics in disguise” (Dear 2011, 165). Although occasionally mentioned in several early modern treatises, such as Tartaglia’s Euclide (1565), Dee’s *Mathematicall Praeface* (1570) and van Roomen’s *Universae Mathesis* (1602), the inclusion of *mixed mathematics* into the Baconian “distribution of Human knowledge” significantly boosted its promotion as a fully-fledged area of research (Jalobeanu 2016, 53). This field clustered several physico-mathematical sciences, of which Bacon offers the following list: “Mixed mathematics [are] *perspective*, *music*, *astronomy*, *cosmography*, *architecture*, *enginery*, and diverse others.” (Bacon 1605, 97). Strikingly enough, this area of learning gathers together several sciences hitherto divided among separate disciplinary groups. According to the traditional classificatory framework prevalent at the end of the middle ages, music and astronomy were part of the quadrivium and regarded as “liberal arts”, while architecture and enginery were classified among “mechanical arts”, a totally different cluster of disciplines. I contend that such a recombination of the mathematical subfields is the result of the famous *thematic reclassification* that occurred in early modern Europe, which has already been long identified in the literature (Kelley 1997).

During the second half of the sixteenth century, a shift gradually occurred in the type of criteria used to classify the sciences, as explained above. While traditionally based on ontological criteria, a growing number of classifications started to involve epistemological considerations (see Fig. 3). As a result, several fields of research previously separated were relocated and grouped according to the new classificatory criteria. In this respect, *mixed mathematics* is archetypal of a field that emerged in such a reconfigured disciplinary framework. During this transition, new epistemological borders have emerged, while the old ontological boundaries have gradually disappeared. Both mechanisms can be straightforwardly observed during the development of *mixed mathematics*. The disparition of the ontological division established by Aristotle between incorruptible entities (celestial bodies, sounds, light, etc.) and corruptible matter (terrestrial bodies) smoothed the gap between the objects previously separated among these categories. In particular, the distinction between physical objects and astronomical entities became challenged: the adoption of epistemological classificatory criteria “obscured this Aristotelian demarcation between terrestrial and celestial objects” (Barker and Chen 2000, S219). Conversely, the introduction of an epistemological border between *pure* and *mixed* mathematics split the quadrivium (including arithmetic, geometry, astronomy and music), hitherto a uniform disciplinary cluster of sciences dealing with incorruptible entities, into two separate groups, rather based on a distinction between rational and empirical knowledge: while *pure* mathematics is now limited to arithmetic and geometry only, *mixed* mathematics includes a set of several empirical sciences. In ontological classifications, such as the one of Hugh of St.-Victor, the quadrivium and the mechanical arts were placed on completely separate branches of the disciplinary tree, while they became *adjacent* in the Baconian system. Such a reorganization has far-reaching consequences: the modalities of contact between the elements involved in the two disciplinary clusters have been radically transformed. *Mixed mathematics* emerged as a result of new interactions “between natural philosophizing and practical mathematics” (Schuster 2017, 51), whose new proximity was due to a thematic reclassification. This contact between mathematical and physical practitioners paved the way to the emergence of our mathematized physics (Sandoz 2018).

## Conclusion

In the light of the above, it appears that several thematic reclassifications have occurred over the centuries, resulting in a deep reshaping of the disciplinary framework of science. More than merely shifting the boundaries between some particular fields, any change in the very criteria underpinning the structure of science affects the organization of research at a global level. The motivations behind a thematic reclassification are multiple, and may often stem from factors extrinsic to the content of science: for instance, I have discussed in the last section how the growing specialization of work in the nineteenth-century society—that pertains to the sociological context of research rather than its content—impelled the adoption of a methodological disciplinary paradigm. As a result, a thematic reclassification is likely to reconfigure the borders between scientific fields in a way that does not necessarily reflect any prior warping in the content of research.

This is especially true since any classification system involves unavoidable logical constraints, as explained above. Hence the successive thematic reclassifications that have occurred over time have entailed a series of recombinations of the objects, knowledge, methods and aims of science, often with the result of outlining new areas of research. Typically, an emerging discipline may apply the method of an old science to the study of a newly discovered object, or recycle an epistemological framework to another purpose. In turn, the development of new research areas has an influence on the disciplinary landscape (Holton 1973; Tondl 1998). Both mechanisms coexist and reinforce each other: thematic reclassifications foster the emergence of new fields, that may in turn hasten the need to revise the disciplinary structure of science. Although the former process, involving the action of a global change on a particular event, is expected to occur more easily than the latter.

In addition, it is interesting to point out that the successive thematic reclassifications that appear on Fig. 3 strikingly echo Kuhn’s chronology of “scientific revolutions”. He located a first turning point in the development of science at the end of the Renaissance, which seems to coincide with the aforementioned shift from ontological to epistemological classification systems: “radical alterations in man’s understanding of nature rapidly followed the publication of Copernicus *De Revolutionibus* in 1543. [This] was an important cause of the general intellectual ferment now known as the scientific revolution.” (Kuhn 1957, 2) Additionally, he acknowledged a second revolution during the first half of the nineteenth century: “Sometime between 1800 and 1850 there was an important change in the character of research […] That change is one facet of a second scientific revolution.” (Kuhn 1961, 190) According to my chart displayed on Fig. 3, the thematic structure of science was substantially reclassified during that period too. Thus, the notion of “thematic reclassification” developed in my paper provides a way to extend the Kuhnian idea that, over the centuries, science undergoes paradigmatic shifts that are conducive to innovative research.

Admittedly, the existence of such Kuhnian “scientific revolutions” has been widely questioned in recent decades. However, fundamental discussions on ruptures and continuities in the development of science remains a topical debate. Amongst the various trends of research carried out on scientific change, several scholars have discussed *ontological ruptures* involving a reclassification of the objects of science (Barker and Chen 2000). Many philosophers have supported the existence of *epistemic ruptures* between theoretical frameworks (Kvasz 1999). The existence of *methodological ruptures* between “incommensurable” experimental practices has sometimes been acknowledged (Hoyningen-Huene 2013; Soler 2008). Finally, the idea of *teleological ruptures* as modifications of the purposes of science has also been examined (Laudan 1984). The historical analysis developed in my paper strongly suggests that these philosophical lines could meet at the level of global thematic reclassifications of the sciences. May the historiographical material I have collected in this regard contribute to the exploration of this important facet of scientific change.

## Footnotes

1 The *Interactive Historical Atlas of the Disciplines* (Sandoz 2020), recently launched at the University of Geneva, and available at: https://atlas-disciplines.unige.ch.

2 I use the word “criterion” in the logical sense, as a “necessary and sufficient condition”.

3 “De tous les principes de classification le plus célèbre est celui de Bacon.[…] Cependant il faut observer qu'il n'est aucune science où l'entendement, l'imagination et la mémoire ne soient employées.” Condorcet (1793a, 767), in the manuscript n.a.fr 4586 f. 55: “Sur le sens des mots *sciences* et *art*, sur les classifications des sciences et des arts”.

4 “Nous avons cru devoir distribuer les sciences d'après les méthodes qu'elles emploient” Condorcet (1792, 466).

5 “La minéralogie et l'application de l'analyse chimique à la connaissance des minéraux ne sont point une même science, mais elles s'exercent sur le même objet, en employant des méthodes différentes.” (Condorcet 1793c, 572).

6 “Il me paraît rationnel de diviser simplement les connaissances d'après le but vers lequel elles tendent.[…] les groupes que l'on obtient en divisant les connaissances humaines d'après cette considération, sont beaucoup plus naturels que ceux que l'on obtient lorsque l’on veut partir, soit des facultés que nous mettons en usage, soit de la nature même des connaissances” (d’Omalius d’Halloy 1834, 2).

7 “Les sciences sont des ensembles de réponses à des questions que se pose l’esprit humain, et les différences les plus radicales entre les sciences résultent de celles qu'il y a entre les questions posées” (Naville 1901, 11).

8 Chaque science est astreinte par la spécialité même de son objet à mettre en œuvre, pour l'explorer, des modes appropriés de recherche. L'ensemble de ces procédés d'investigation constitue sa méthode.[…] Les mêmes considérations qui induisent à séparer dans la nature les catégories de faits obligent à les étudier de différentes façons. Il y a donc lieu d'établir autant de méthodes que nous avons institué de sciences.” (Bourdeau 1882, I, 36–37).

9 Little is known about George Albert Cogswell, born in Port Williams (Canada) in 1865 before moving to the United States for his studies at Cornell University, where he obtained a PhD in 1898 for a thesis in philosophy.

10 “La classification des sciences peut se ramener à une *table à double entrée*. Les sciences objectivement analogues se trouveront sur la même ligne *horizontale*, et celles qui se ressemblent par leur but, sur la même *verticale*.” (Saint-Hilaire 1854, I, 261).

11 Available at: https://atlas-disciplines.unige.ch. This paper is based on the version of September 2020, archived for future reference and accessible through the following permalink: https://purl.org/atlas-disciplines/09-2020.

12 A tenacious misunderstanding places *mixed mathematics* in the direct continuity of Aristotle’s so-called “subordinate sciences”. Such an assumption is misleading: as rightly noted by Capecchi (2017, 156), Aristotle never considered “subordinate sciences” *tout court*, but only with respect to another field, in the sense of an ordering relation. The transition from a relation of order (subordination) to an intersection (mixed) is most probably a rhetorical strategy put forward by early promoters of *mixed mathematics* in a context of Aristotelianism.
